# The clinical efficacy of artemether/lumefantrine (Coartem^®^)

**DOI:** 10.1186/1475-2875-8-S1-S5

**Published:** 2009-10-12

**Authors:** Michael Makanga, Srivicha Krudsood

**Affiliations:** 1 European & Developing Countries Clinical Trials Partnership (EDCTP), Francie van Zijl Drive, Parow, P.O. Box 19070, Tygerberg 7505, Cape Town, South Africa; 2 WHO CC for Clinical Management of Malaria, Faculty of Tropical Medicine, Mahidol University, 420/6 Rajavithee Road, Rajathewee, Bangkok 10400, Thailand

## Abstract

Current World Health Organization (WHO) guidelines for the treatment of uncomplicated falciparum malaria recommend the use of artemisinin-based combination therapy (ACT). Artemether/lumefantrine is an ACT prequalified by the WHO for efficacy, safety and quality, approved by Swissmedic in December 2008 and recently approved by the USA FDA. Coartem^® ^is a fixed-dose combination of artemether and lumefantrine. Its two components have different modes of action that provide synergistic anti-malarial activity. It is indicated for the treatment of infants, children and adults with acute, uncomplicated infection due to *Plasmodium falciparum *or mixed infections including *P. falciparum*. A formulation with improved palatability has been developed especially for children (Coartem^® ^Dispersible), which rapidly disperses in a small amount of water for ease of administration.

The efficacy of the six-dose regimen of artemether/lumefantrine has been confirmed in many different patient populations around the world, consistently achieving 28-day PCR (polymerase chain reaction)-corrected cure rates of >95% in the evaluable population, rapidly clearing parasitaemia and fever, and demonstrating a significant gametocidal effect, even in areas of widespread parasite resistance to other antimalarials.

## Background

The rising threat of *Plasmodium falciparum *resistance to monotherapies prompted the World Health Organization (WHO) 2006 guidelines for the treatment of malaria to recommend that combinations of antimalarials be used to treat malaria caused by *P. falciparum *[1]. Since artemisinin derivatives are the only class of anti-malarial agents to which resistance outside the Thai-Cambodia border region has not been reported *in vivo *[1], the guidelines specifically recommend the use of artemisinin-based combination therapy (ACT) [1]. WHO criterion for adequate efficacy of ACT in malaria is the achievement of an average cure rate of ≥ 95% in clinical trials [1].

Artemisinin derivatives have the most potent and rapid onset of anti-parasitic activity of any anti-malarial drug available today and are active against all *Plasmodium *species that infect humans. Importantly, they allow more parasite clearance than any other anti-malarial drug (parasite numbers can be reduced by a factor of 10^5 ^per asexual cycle, compared with 10^2^-10^3 ^with other anti-malarial drugs) [2]. When combined with efficacious anti-malarials with slower elimination rates, such as lumefantrine, shorter courses of treatment (three days) become effective [2].

The combination of artemether (an artemisinin derivative) and lumefantrine in a 1:6 ratio was the first fixed-dose ACT to meet the WHO's prequalification criteria for efficacy, safety and quality [3]. Artemether/lumefantrine (AL), as Coartem^®^, now comprises nearly 75% of the 100 million or so ACT treatments used each year [4]. Coartem^® ^was approved by Swissmedic in 1999 and was recently approved by the USA FDA [5]. Coartem^® ^Dispersible was approved by Swissmedic in December 2008. More than 40 malaria-endemic countries in sub-Saharan Africa have rapidly scaled up malaria prevention and treatment and now recommend the use of ACT as first-line treatment for uncomplicated falciparum malaria [6]. Both artemether and lumefantrine are blood schizonticides with complementary pharmacokinetics and dissimilar modes of action, and hence provide synergistic anti-malarial activity [7]. Artemether is rapidly eliminated from plasma with a half-life of two to three hours, whereas lumefantrine is eliminated more slowly with a half-life of three to six days and provides a high long-term cure rate after a short treatment course [7]. The combination thus provides rapid clearance of parasitemia and most malaria-related symptoms, coupled with prevention of recrudescence.

AL tablets have been included on the WHO model list of Essential Medicines since March 2002 and on the first WHO Model List of Essential Medicines for Children since October 2007 [8,9].

The six-dose AL regimen is currently the approved treatment regimen for acute, uncomplicated *P. falciparum *malaria in adults and paediatric patients with a body weight ≥ 5 kg, irrespective of the immune status of the patients to *P. falciparum *and of the local multidrug resistance situation, in the majority of the 83 countries in Africa, Asia, Europe and Latin America where the drug is registered.

The objective of this paper is to review the literature investigating the drug's clinical efficacy. In this respect, it is important to note that early clinical studies used a four-dose AL regimen (two doses per day for two days).

However, because this regimen did not provide optimal efficacy in some areas, such as Thailand, where multi-drug resistant *P. falciparum *malaria is prevalent, it was changed to its current six-dose format (three-day course: dosing at 0, 8, 24, 36, 48 and 60 hours) in 1997. This article will focus on data involving the six-dose regimen only. In addition to the key clinical trials that support the drug's indication for the treatment of uncomplicated *P. falciparum *malaria, over 40 independent trials have produced data supporting the excellent efficacy of this ACT.

### Study design considerations

The active comparators used in the controlled studies summarized below were the standard therapies for each country as recommended by the WHO at the time. Placebo-controlled studies are not practical for ethical reasons because untreated *P. falciparum *malaria may progress rapidly, with a potentially fatal outcome. All comparative studies were randomized, and double-blind designs were used where practical.

### Efficacy evaluations

The key criterion used to assess the efficacy of an anti-malarial agent is the elimination of malaria parasites, which in turn leads to resolution of symptoms such as fever. Unless otherwise stated, the primary efficacy endpoint in the studies outlined below was the 28-day parasitological cure rate. This describes the proportion of patients with clearance of asexual parasitaemia within seven days of initiating study treatment without recrudescence at day 28, based on blood smears. In most studies, the 28-day cure rate was also corrected by polymerase chain reaction (PCR) to differentiate between recurrence of the initial infection and a new infection. This is particularly important in highly endemic regions, as is the case in many parts of sub-Saharan Africa, where reinfection is common.

Several secondary efficacy endpoints were also evaluated. These included: 7-day, 14-day, and/or 42-day parasitological cure rates; fever clearance time (FCT) in patients who had fever at baseline; parasite clearance time (PCT) and gametocyte clearance time (GCT).

The evaluable population included all patients with confirmed *P. falciparum *malaria who received at least one dose of study drug and had parasite counts performed at the pre-specified time points, including day 28, or who discontinued due to unsatisfactory therapeutic effect.

The primary efficacy endpoint was also evaluated in the modified intent-to-treat (mITT) population, which included all patients with confirmed *P. falciparum *malaria who received at least one dose of study drug. Since patients who did not have a parasite count performed at day 7 or day 28 were classified as a treatment failure in the mITT analysis, the evaluable population analysis gives the most clinically relevant result as it better reflects the true activity of the drug (i.e. it avoids classifying as treatment failure patients who did not have a parasite count performed but who may have been cured).

## Efficacy of AL studied in children and adults

Six key clinical studies, summarized in Table 1[10-15], have been conducted to evaluate the efficacy of the six-dose AL regimen in a range of patient populations and geographic regions with varying levels of drug resistant *P. falciparum *and malaria endemicity. In all studies, patients were either non-immune or semi-immune to *P. falciparum*. Some of the studies included other anti-malarial drugs or combinations as active comparators and some also allowed the inclusion of patients with mixed infections that included *P. falciparum *at baseline.

**Table 1:** Clinical studies evaluating the efficacy and safety of the six-dose regimen of artemether/lumefantrine

**Study number**	**A025 **[10]	**A026**[11]	**A028**[12]	**A2403**[13]	**B2303**[14]	**A2401**[15]
Design	Randomized double-blind	Randomized open-label	Randomized open-label	Open-label	Randomized investigator blind	Open-label
Comparator	Four-dose regimen	MAS^a^	MAS^a^	-	Dispersible formulation	-
Patients	Adults & children (>2 years)	Adults & children (≥ 2 years)	Adults & adolescents (>12 years)	Infants & children (5 to 25 kg)	Infants & children (5 to <35 kg)	Adult non-immune travellers
N AL/total Geography	120/359^b^	150/200	164/219	310	452/899	165
	Thailand	Thailand	Thailand	Kenya	Kenya	EU
				Tanzania	Tanzania	Colombia
				Nigeria	Mali	
					Benin	
					Mozambique	
28-day PCR-corrected cure rate (evaluable pts)	96.9%	97.7%	95.5%	96.7%	97.8%	96.0%
Median time to fever clearance (h) (mITT pop^n^)	35 (n = 59)	22 (n = 87)	29 (n = 76)	7.8 (n = 309)	7.8 (n = 311)	36.5 (n = 100)
Median time to parasite clearance (h) (mITT pop^n^)	43.6 (n = 118)	48 (n = 149)	29.3 (n = 164)	24.0 (n = 310)	34.9 (n = 452)	41.8 (n = 162)

^a^Study was not designed to compare AL with mefloquine + artesunate; ^b^AL over 60 hours; ^c^28-day cure rate was a secondary endpoint. Abbreviations: AL, artemether/lumefantrine; MAS, mefloquine + artesunate.

### Study AO25

This randomized, double-blind study enrolled 359 adults and children above two years of age with uncomplicated *P. falciparum *in two centres in Thailand. It compared the AL four-dose regimen to two different six-dose regimens, one administered over 60 hours and one administered over 96 hours [10].

**Figure 1:**
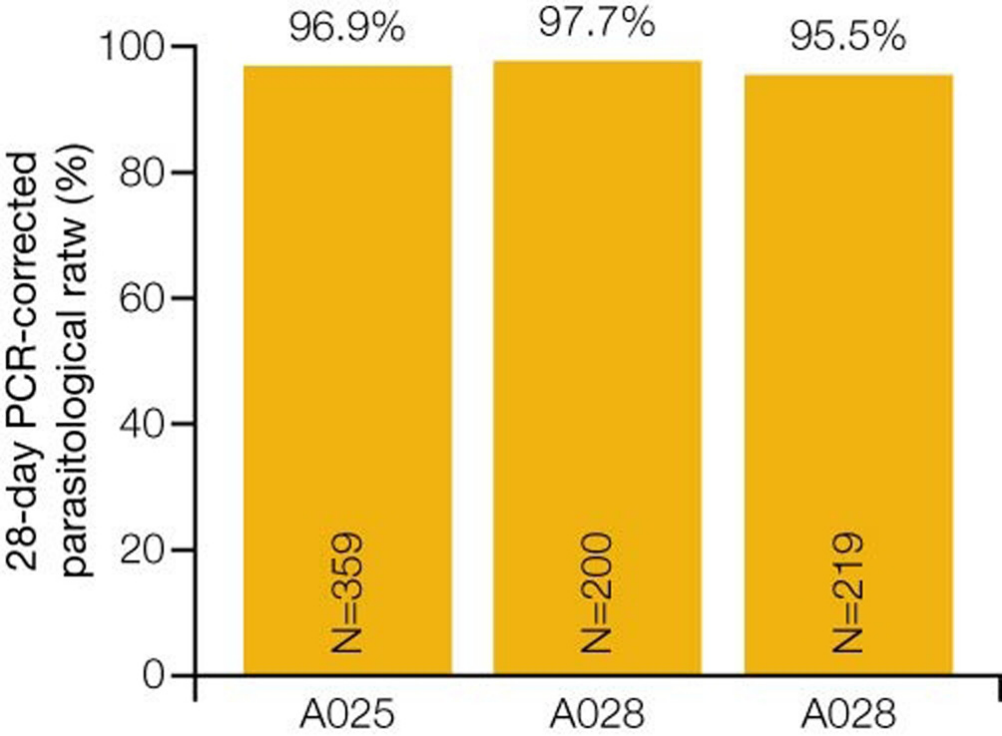
**28-day PCR-corrected cure rate for artemether/lumefantrine in Studies A025,A026 and A028 **[10-12].

AL achieved a 28-day PCR-corrected cure rate of 96.9% in the evaluable population (Figure 1) [10]. All patients cleared peripheral parasitaemia rapidly - the median parasite clearance time was 44 hours [10]. Median fever clearance time was 35 hours, and no patient developed gametocytaemia following treatment [10].

Since the efficacy was similar between the 96 and 60-hour six-dose regimens, the 60-hour regimen was chosen for further development to facilitate better compliance with a shorter treatment duration.

### Studies A026 and A028

These two randomized, open-label studies were both conducted in Thailand and included mefloquine plus artesunate (MAS) as an active control arm [11,12].

Study 026 enrolled patients ≥ 2 years of age [11], and Study 028 enrolled patients >12 years of age [12]. The open-label design for both studies was chosen because of the excessive number of placebo tablets (24) patients would have had to take if a double-blind/double-dummy design had been used.

In Study 026, 150 patients received AL and 50 patients received mefloquine and artesunate. Patient characteristics were comparable between treatment groups, and treatment comparisons were not affected by a difference in baseline parasite density. The 28-day PCR-corrected cure rates were >95% in the evaluable population for both treatment groups (Figure 1). The median fever and parasite clearance times were identical in the two treatment groups [11].

In Study 028, 164 patients received AL and 55 patients received MAS. Patient characteristics were similar to those seen in Study 026 and were comparable across treatment groups, except for parasite density which was slightly lower than in Study 026 [11,12].

Once again, the 28-day cure rates were >95% in the evaluable population and were comparable in both treatment groups (Figure 1). The lower boundary of the confidence interval was greater than 85%. Median fever and parasite clearance times were in the range of 20 to 30 hours in both groups [11,12].

### Study A2403

This was an open-label non-comparative study performed in collaboration with the WHO. It was designed to examine the safety and efficacy of AL in 310 infants and young children (5-25 kg body weight) in sub-Saharan Africa (Kenya, Nigeria, and Tanzania) [13]. In the absence of an ethical comparator agent for paediatric patients with a body weight of 5 to <10 kg at the time of the study, it was a single-arm study. The median age was 24 months, ranging from two months to 10 years. AL tablets were crushed and mixed with sterile water to facilitate administration to those children who were unable to swallow whole tablets [13]. In all cases, treatment was given after feeding.

The overall 28-day cure rate, corrected for reinfection, was 93.9% for the intent-to-treat population [13]. The median time to parasite clearance ranged from 24 to 36 hours, with 98.4% of patients achieving parasite clearance within 48 hours of their first dose [13]. The median time to fever clearance was less than eight hours [13], and none of the children had gametocytes after day 14 [13].

### Study B2303

To facilitate administration of AL to children, a sweet-tasting dispersible formulation has been developed. The primary objective of Study B2303, which enrolled 899 African children (5-<35 kg body weight), was to demonstrate non-inferiority of the dispersible tablet to the crushed tablet based on the 28-day PCR-corrected cure rate in the evaluable population. The median age of children enrolled was 36 months, ranging from two months to 12 years, and the median body weight was 13 kg [14].

**Figure 2:**
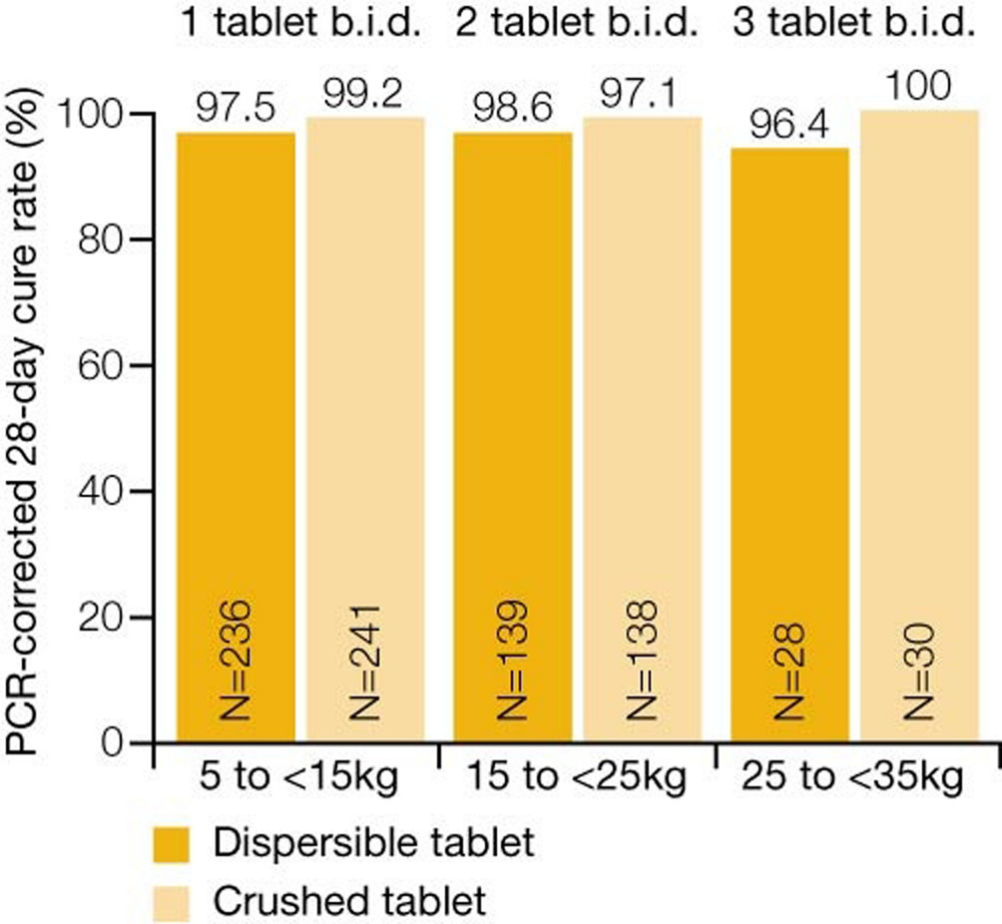
**Efficacy of dispersible AL formulation across body weight groups **[14].

**Figure 3:**
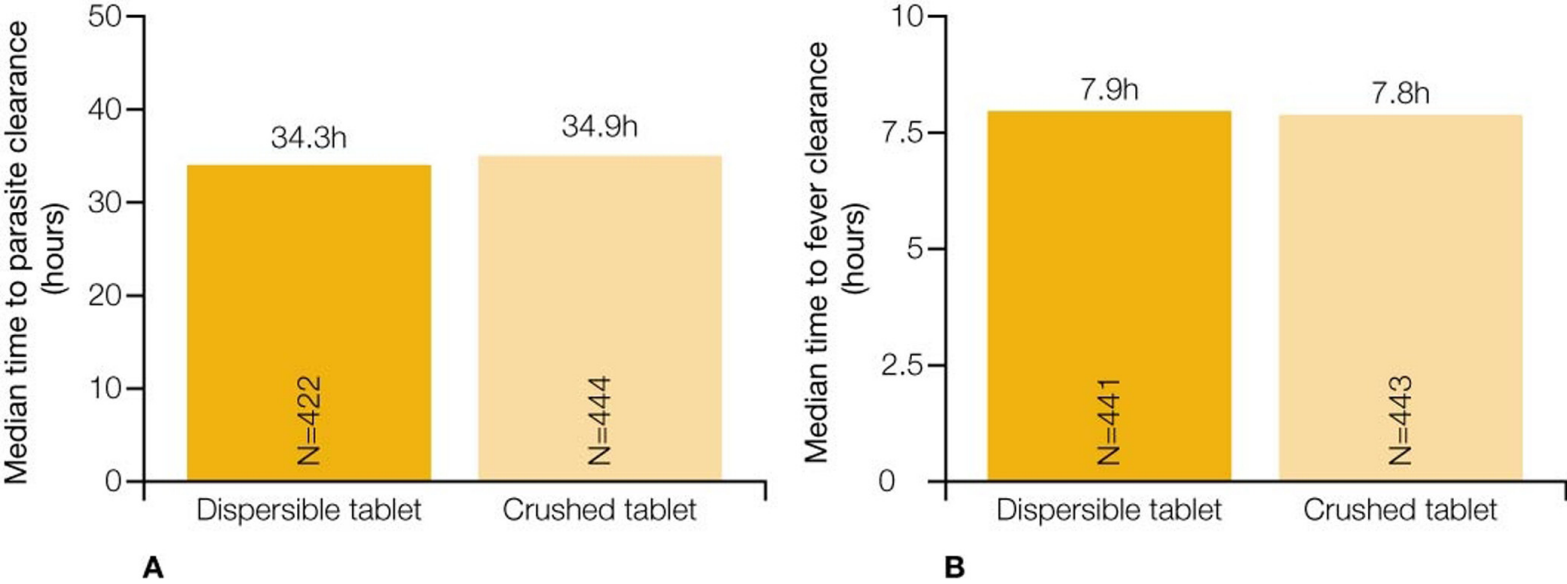
**Median time to (A) parasite clearance and (B) fever clearance for dispersible artemether/lumefantrine formulation and crushed tablets **[14].

In the evaluable population, the 28-day PCR-corrected cure rate for the group receiving crushed tablets was 98.5%, compared with 97.8% for the group receiving the dispersible formulation of AL (Coartem^® ^Dispersible) [14]. The PCR-adjusted cure rates for the two groups were also comparable at day 14 and day 42 [14]. Notably, the cure rates were comparable across body weight groups (Figure 2) [14]. In addition, there were no significant differences between those receiving the dispersible formulation and those receiving crushed tablets with regard to median time to parasite clearance (Figure 3A) and median time to fever clearance (Figure 3B) [14].

### Study A2401

This study was designed to assess the efficacy of AL in non-immune populations (e.g. travellers). To date, it is the largest study investigating a drug for the treatment of malaria in travellers. Because of practical considerations related to the availability of patients, it was an open-label, single-arm study. A total of 165 patients who had been diagnosed with non-endemic malaria were enrolled from Europe and non-malarious areas of Colombia. Non-immune patients were defined as those who had not spent the first five years of their life, nor the five years prior to study entry, in a malaria-endemic area, and had not had acute *P. falciparum *malaria diagnosed during the past 5 years.

**Figure 4:**
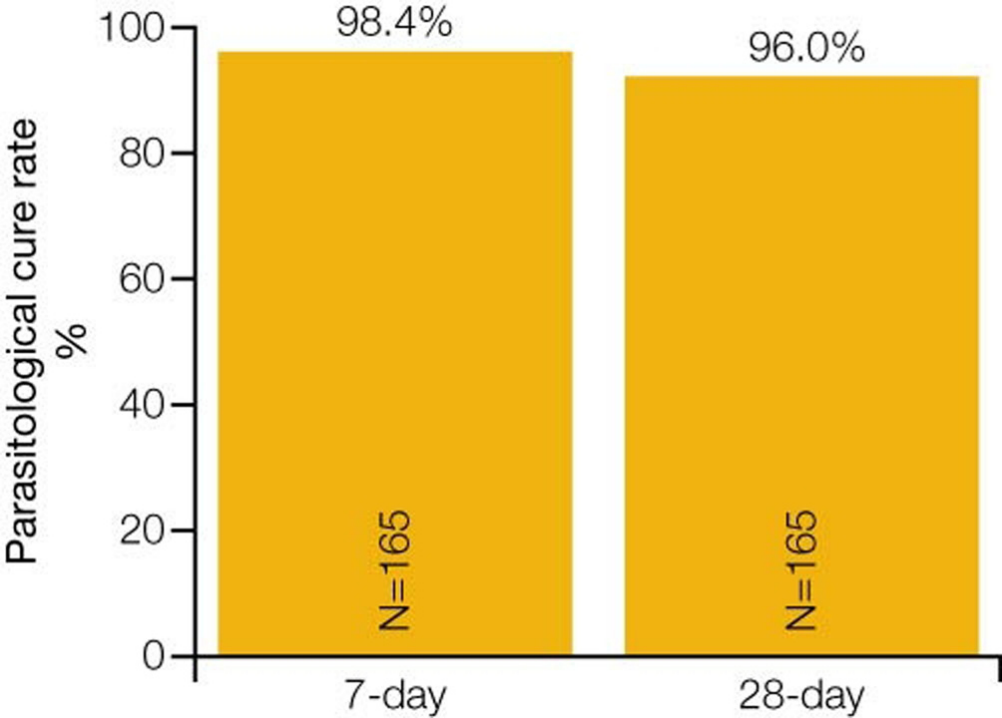
**28-day PCR-corrected cure rate for AL in Study A2401 **[15].

A high 28-day cure rate of 96% (both uncorrected and PCR-corrected) was observed in the evaluable population (Figure 4) [15]. The median time to parasite clearance was 41.5 hours and the median time to fever clearance was 36.8 hours [15].

A total of 599 adult patients (>16 years) and 877 children (<16 years) were treated with the six-dose AL regimen in the above studies. The 28-day PCR-corrected cure rate in evaluable patients consistently met WHO criteria for efficacy (>95%) in both adults and children. Rapid clearance of parasitaemia and fever was also evident, although noticeably quicker in children.

## Comparative results from independent studies

A large number of independent studies have compared the efficacy of AL with other anti-malarial treatments. These studies conclusively show that AL is highly efficacious and at least as effective as other ACTs and combinations of antimalarials over a wide range of geographical areas and in a variety of populations. A summary of these trials is presented in Tables 2 and 3.

**Table 2:** Published studies with the six-dose AL regimen in Africa

**Authors**	**Country**	**Design and patient population**	**Comparator(s) No. of patients**	**Results**
Bukirwa et al 2006 [16]	Uganda	Randomized, single-blind, single centre in children (1-10 years)	AL (n = 208) ASAQ (n = 211)	Primary efficacy outcome was the 28-day risk of recurrent symptomatic malaria (AL = 27%;ASAQ = 42%) and recurrent parasitaemia (AL = 51%;ASAQ = 66%) both unadjusted for re-infection (both p = 0.001). When corrected for re-infection using PCR, the risks for both recurrent symptomatic malaria and parasitaemia were 0% for ASAQ and 1% for AL (equivalent to PCR-adjusted 28-day parasitological cure rates of 100% and 99%, respectively), illustrating the high rate of re-infection in the area studied.
Dorsey et al 2007 [17]	Uganda	Single-blind, randomized, single centre in children (1-10 years)	AL (n = 105) AQSP (n = 111) ASAQ (n = 113)	PCR-corrected 28-day cure rates of 99% with AL, 95.4% with ASAQ and 85.9% with AQSP. The differences between AL and AQSP, and between ASAQ and AQSP were statistically significant (p < 0.001 and p = 0.08, respectively). Clearance of fever, sexual parasites and gametocytes were similar in the AL and ASAQ groups but slower in the AQSP group.
Gürkov et al 2008 [18]	Ethiopia	One centre, comparative in adults and children >5 years of age	AL (n = 30) Quinine (n = 5) Atovaquone/Proguanil (n = 32)	At 28-days, there were no treatment failures in the AL group, but the PCR-confirmed recrudescence rates in the quinine and atovaquone/proguanil groups were 9% and 6%, respectively.
Kabanywanyi et al 2007 [19]	Tanzania	Randomized, open-label, 2-centre in children (6-59 months)	AL (n = 99) ASAQ (n = 76)	28-day PCR-corrected rates of adequate clinical and parasitological response were 100% for AL and 93.8% for ASAQ. Late parasitological failures, in all cases due to re-infection rather than recrudescence, occurred in 12% and 29% of each group, respectively.
Kamya et al 2007 [20]	Uganda	Single-blind, randomized, single centre in children (6 months to 10 years)	AL (n = 210) DP (n = 211)	PCR-corrected risk of recurrent parasitaemia at Day 28 was significantly lower with DP than with AL (1.9% vs. 8.9%); this was also the case at Day 42 (6.9% vs. 16%). However, due to the complexity of infection in this area of very high transmission, leading to difficulty in distinguishing between new and recrudescent infections, the risks of recrudescence are probably overstated. Times to clearance of fever and parasites were similar in the two treatment groups, although DP was associated with better control of gametocytes.
Mårtensson et al 2005 [21]	Zanzibar	Multicenter, randomized, open-label in children (6-59 months)	AL (n = 200) ASAQ (n = 208)	PCR-corrected 28-day and 42-day parasitological cure rates of 97% and 92%, respectively, for AL and 91% and 88%, respectively, for ASAQ (p = 0.001 at 28 days and p = 0.045 at 42 days). Parasite and fever clearance were rapid with both treatments, and gametocyte carriage was low in both groups.
Mårtensson et al 2007 [22]	Tanzania	Randomized, single centre, open-label study in children	AL (n = 50) SP (n = 56)	PCR-corrected 42-day parasitological cure rates were 98% or 94% in the AL group (depending on whether standard or enhanced PCR was used); corresponding figures in the SP group were 70% and 66%, respectively.
Mohamed et al 2006 [23]	Sudan	2-centre, open-label, treatment assigned by centre, in children and adults	AL (n = 72) ASSP (n = 71)	In this area of low malaria transmission both AL and ASSP were associated with adequate clinical and parasitological response rates of 100% at Day 28.
Mukhtar et al 2007 [24]	Sudan	Randomized, single centre, open-label study in children & adults	AL (n = 80) ASSP (n = 77)	PCR-corrected rates of adequate clinical and parasitological response of 93.4% for ASSP and 91.3% for AL. The treatment regimen used was not clear.
Mutabingwa et al 2005 [25]	Tanzania	Randomized, single centre, open-label study in children	AL (n = 519) ASAQ (n = 515) AQSP (n = 507) AQ (n = 270)	PCR-corrected 28-day parasitological cure rates were 51.6% for AQ, 65.5% for AQSP, 88.8% for ASAQ, and 97.2% for AL. Recruitment to the AQ group was stopped early by the trial's data and safety monitoring board due to a high treatment failure rate.
Yeka et al 2008 [26]	Uganda	Randomized, one centre, single-blinded, in children aged 6 months to 10 years	AL (n = 227) DP (n = 234)	At 42 days there was no statistically significant difference in the risk of recrudescence (5.8% for AL vs. 2.0% for DP; risk difference = 3.8%, 95% CI 0.2-7.8%), although recurrent parasitaemia (uncorrected for re-infection) was more frequent with AL (33.2% vs. 12.2% for DP).
Mulenga et al 2006 [27]	Zambia	Randomized, open-label, multicentre, in adults	AL (n = 485) SP (n = 486)	AL was associated with significantly faster clearance of fever, parasitaemia and gametocytes than SP, and a higher Day 45 PCR-corrected cure rate (94.6% vs. 80.7%, p < 0.001).
Toovey 2008 [28]	Mozambique	Non-comparative, open-label, single centre in adults	AL (n = 54)	28-day parasitological cure rate of 100%.
Adjei et al 2008 [29]	Ghana	Randomized, open label	AL (n = 111) AS+AQ (n = 116)	For the AL group, adequate clinical and parasitological response was reported in 97.1% patients at Day 14 and 94.2% at Day 28; corresponding figures for ASAQ were 98.2% and 95.3%.
Falade et al 2008 [30]	Nigeria	Randomized, open label, single centre in children (6 months to 10 years)	AL (n = 66) ASAQ (n = 66)	PCR-corrected 28-day parasitological cure rates of 100% for AL and 98.4% for ASAQ.
Faye et al 2007 [31]	Senegal	Randomized, open-label, multicentre in children and adults	ASAQ (n = 360) AQSP (n = 161) MAS (n = 145) AL (6 dose) (n = 149) AL (four-dose) (n = 140)	PCR-corrected 28-day parasitological cure rates were 100% for all treatments other than AL four-dose (96.4%). Parasite clearance was observed to be more rapid with all ACTs than with AQSP. Initial reduction of gametocyte carriage also appeared to be more rapid with the ACTs than with AQSP, although all patients were free of gametocytes by day 21.
Koram et al 2005 [32]	Ghana	Multicentre, randomized, open-label in children (6-59 months)	AL (n = 51) CQ (n = 36) SP (n = 27) ASAQ (n = 54)	PCR-corrected 28-day cure rates of 25% for chloroquine, 60% for SP, 100% for ASAQ, and 97.5% for AL.
Meremikwu et al 2006 [33]	Nigeria	Randomized, open-label, single centre, in children (6-59 months)	AL (n = 60) ASAQ (n = 59)	Both treatments highly effective with similar rates of adequate clinical and parasitological response, early treatment failure, late clinical failure and late parasitological failure at 14 days.
Owusi-Agyei et al 2008 [34]	Ghana	Randomized, open label in children aged 6 months to 10 years	AL (n = 223) ASAQ (n = 220) ASCD (n = 178)	Per-protocol analysis showed a lower PCR-corrected parasitological and clinical failure rate at day 28 in the ASAQ group (6.6%) compared with the AL group (13.8%) or ASCD group (13.8%).
Sagara et al 2006 [35]	Mali	Randomized, single centre, open-label study in children (≥ 6 months) and adults	AL (n = 303) AS plus sulphamethoxypyrazine plus pyrimethamine (n = 303)	28-day PCR-corrected parasitological cure rates were 100% for AS plus sulphamethoxypyrazine plus pyrimethamine, and 99% for AL. Gametocyte clearance was similar in the two treatment groups.
Sowunmi et al 2007 [36]	Nigeria	Randomized, open-label, single centre, in children (≤ 10 years)	AL (n = 90) AQ plus sulphalene plus pyrimethamine (n = 91)	PCR-corrected parasitological cure rates at 42 days were 93.3% for AL and 98.9% for AQ plus sulphalene plus pyrimethamine.
Sutherland et al 2005 [37]	Gambia	Randomized, single centre, single-blind, in children (1-10 years)	AL (n = 406) CQSP (n = 91)	PCR-corrected 28-day cure rates were 96.1% for AL and 91.1% for CQSP. AL-treated patients were statistically significantly less likely to carry gametocytes at Day 28 than those who received CQSP (8% vs. 49%, p < 0.0001), and carriers in the AL group harboured gametocytes at lower densities, for shorter periods (0.3 d vs. 4.2 d, p < 0.0001) and were less infectious to mosquitoes at day 7 (p < 0.001) than carriers in the CQSP group.
Zongo et al 2007 [38]	Burkina Faso	Multicentre, randomized, open-label in children (6 months to 10 years)	AL (n = 261) AQSP (n = 260)	The crude (uncorrected for re-infection) risk of recurrent malaria at 28 days was significantly higher with AL than AQSP (10.2% vs. 1.7%, p < 0.0001); PCR correction gave risks of recurrent malaria of 1.2% and 0.4%, with the between-group difference not statistically significant.
Zongo et al 2007 [39]	Burkina Faso	Multicentre, randomized, open-label in children (≥ 6 months)	AL (n = 188) AQSP (n = 184) DP (n = 187)	PCR-corrected risks of re-infection at Day 28 were AL, 3.4%; DP, 2.2% and AQSP, 3.9%.
Fanello et al 2007 [40]	Rwanda	Randomized, open-label, 2-centre in children (12-59 months)	AL (n = 251) AQSP (n = 249)	AL was associated with a statistically significantly higher rate of PCR-adjusted adequate clinical and parasitological response at Day 28 than AQSP (96.8% vs. 79.4%, p < 0.0001). Gametocyte carriage was significantly lower in the AL group at all post-baseline time points.
Guthman et al 2006 [41]	Angola	Randomized, open-label, single centre in children (6-59 months)	AL (n = 61) ASAQ (n = 64)	PCR-corrected 28-day parasitological cure rates were 100% for both AL and ASAQ.
Ndayiragije et al 2004 [42]	Burundi	Multicentre, randomized, open-label in children (<5 years)	AL (n = 142) ASAQ (n = 153)	14-day rates of adequate clinical and parasitological response were similar between treatment groups:AL = 99.3%;ASAQ = 95.3%. Effects on gametocyte carriage were also similar between treatments.
van den Broek et al 2006 [43]	Republic of Congo	Randomized, single centre, open-label study in children (6-59 months)	AL (n = 106) ASAQ (n = 101) ASSP (n = 91)	PCR-corrected 28-day parasitological cure rates were 100% for AL, 98.5% for ASAQ and 90.1% for ASSP. The differences in cure rates between AL and ASSP, and between ASAQ and ASSP, were statistically significant. Clearance of asexual parasites, gametocytes and fever were rapid in all three treatment groups.

AL:Artemether/lumefantrine; CQ: Chloroquine; SP: Sulphadoxine plus pyrimethamine; CQSP: Chloroquine plus sulphadoxine plus pyrimethamine;AS: Artesunate;AQ:Amodiaquine;ASAQ:Amodiaquine plus artesunate;AQSP:Amodiaquine plus sulphadoxine-pyrimethamine; DP: dihydroartemisinin plus piperaquine; MAS: mefloquine plus artesunate

**Table 3:** Published studies with the six-dose artemether/lumefantrine regimen in Asia

**Authors**	**Country**	**Design and patient population**	**Comparator(s) No. of patients**	**Results**
*South-East Asia*				
Krudsood et al 2003 [45]	Thailand	Randomized, open-label, single centre in adults	AL (n = 41) DNP (n = 89)	The 28-day parasitological cure rates (not PCR corrected) were 99% for DNP and 97% for AL.
Rojanawatsirivej et al 2003 [46]	Thailand	Open-label, multicentre, adults & children (10-74 years), treatment by area (all treatment groups also received primaquine)	AL (n = 33) MAS (n = 199) Mefloquine (n = 318)	All 33 (100%) patients treated with AL plus primaquine had adequate clinical response (i.e. parasitological cure at Day 28), compared with 86.3% of the 80 patients who received mefloquine plus primaquine.
Stohrer et al 2004 [47]	Laos	Randomized, open-label, single centre, in adults & children (≥ 10 kg)	AL (n = 53) MAS (n = 55)	PCR-corrected cure rates at Day 42 were 93.6% for AL and 100% for MAS. The difference between treatment groups was not statistically significant. Day 28 cure rates were identical to those at Day 42.
Mayxay et al 2004 [48]	Laos	Randomized, open-label, single centre, in adolescents and adults (12-19 years)	AL (n = 110) MAS (n = 110) CQSP (n = 110)	42-day PCR-corrected parasitological cure rates were 97% for AL, 100% for MAS and 93% for CQSP. The difference in cure rates between MAS and CQSP was statistically significant.
Ratcliff et al 2007 [49]	Indonesia	Randomized, open-label, 2-centre in children (body weight ≥ 10 kg) and adults with *P. falciparum*, *P. vivax*, or mixed infections	AL (n = 387) DP (n = 387)	For *P. falciparum *infections, rates of recrudescence at 42 days (PCR-corrected) were 4.7% with AL and 4.1% with DP.
Hutagalung 2005 [50]	Thailand	Randomized, open-label, 2-centre, in children (>10 kg) and adults	AL (n = 245) MAS (n = 245)	Both treatments were associated with rapid clearance of fever and parasitaemia. 42-day parasitological cure rates were 98.8% for AL and 96.3% for MAS.
*South Asia*				
van den Broek et al 2005 [51]	Bangladesh	Randomized, open-label, single centre, in adults & children (≥ 1 year)	AL (n = 121) MAS (n = 121) CQSP (n = 122)	Day 42 PCR-corrected cure rates were 62.4% for CQSP, 100% for MAS and 97.1% for AL. The cure rate in the CQSP group was statistically significantly lower than that in the other treatment groups.
Haque et al 2007 [52]	Bangladesh	Open-label, non-comparative, 2-centre in adults (≥ 18 years)	AL (n = 67)	Rapid fever and parasite clearance, and PCR-corrected 28- and 42-day parasitological cure rates of 98.3% and 94.3%, respectively.
Thapa et al 2007 [53]	Nepal	Randomized, open-label, single centre, in adults & children (>5 years)	AL (n = 66) SP (n = 33)	The 28-day PCR-corrected parasitological cure rate for AL was 100%, compared with 87.9% in the SP group (p = 0.011).

^1^Includes 2 MAS regimens (mefloquine at 25 mg/kg, n = 153, and 15 mg/kg, n = 46);AL: artemether/lumefantrine; SP: sulphadoxine plus pyrimethamine; CQSP: Chloroquine plus sulphadoxine pyrimethamine; DP: Dihydroartemisinin plus piperaquine; DNP: dihydroartemisinin, napthoquine and trimethoprim; MAS: mefloquine plus artesunate

### Studies in Africa

In studies reported from Africa, summarized in Table 2[16-43], the six-dose AL regimen appeared to be at least as effective as most other forms of ACT included in comparative studies and was generally more effective than other, non-ACT, comparators. In the few studies where comparators were associated with lower risks of treatment failure than AL, the studies were typically conducted in areas with very intense malaria transmission, and the comparators included anti-malarials with a long half-life, which could prevent re-infection late in the study. Indeed, the lower crude failure rates with the comparators in these few studies are the result of lower re-infection rates - recrudescence rates were generally similar for AL and comparators.

Several of the publications report the potent effects of ACT treatment on gametocyte carriage. One study investigated gametocyte carriage in detail and it was found that in AL-treated patients, not only was the rate of carriage of gametocytes significantly reduced as compared with that in patients receiving CQSP, but the carriage time of gametocytes and the infectivity of the gametocytes for mosquitoes were also significantly decreased [37]. These findings potentially have considerable implications for public health, given the possibility of reducing transmission of malaria by extensive use of ACT.

### Supervised vs unsupervised AL in acute, uncomplicated falciparum malaria

Piola *et al *[44] conducted a randomized trial to compare the efficacy, safety, and pharmacokinetics of AL when given in a supervised (all doses observed with fatty food intake; n = 313) or unsupervised (first dose supervised followed by outpatient treatment with nutritional advice; n = 644) setting to patients of all ages (weight >10 kg) with acute, uncomplicated falciparum malaria in Mbarara, Uganda. The primary endpoint was 28-day, PCR-adjusted, parasitological cure rate.

28-day cure rates were very similar (100% in both groups in an evaluable patient analysis). The only difference between supervised and unsupervised treatment was that blood lumefantrine levels in the unsupervised patients were statistically significantly lower (p < 0.001) at Days 3 and 7.

### Studies in Asia

The studies reported from South-East Asia, summarized in Table 3[45-53], show that in general AL is associated with similar efficacy to other forms of ACT in the treatment of *P. falciparum *malaria, with high parasitological cure rates and rapid clearance of parasitaemia and resolution of fever. In one study in which *P. vivax *infections were included, the comparator ACT (DP) was significantly more effective in terms of reducing the risk of recurrent *P. vivax *infection, probably due to the long half-life of piperaquine offering protection against re-infection for longer than lumefantrine: in the same study, PCR-corrected *P. falciparum *parasitological cure rates were almost identical for AL and DP [50].

Studies from South Asia show high parasitological cure rates and rapid clearance of fever and parasitaemia with the six-dose AL regimen, comparable with those for MAS in the one study where this combination was used as a comparator. AL was more effective than non-ACT comparators in these studies.

### Meta-analysis

A meta-analysis of 32 published randomized studies performed predominantly in Africa but also in South America and Asia has evaluated the efficacy of a number of different forms of ACT using a Bayesian random effects approach. The analysis showed that AL was one of the most effective ACT with a 28-day PCR-corrected parasitological cure rate of 97.4% (Table 4) [54].

**Table 4:** Results of a meta-analysis comparing the efficacy of several anti-malarial combinations [54]

**Treatment combination**	**28-day PCR-corrected cure rate (%)**
Artemether/lumefantrine (AL)	97.4
Mefloquine + artesunate (MAS)	96.9
Amodiaquine + artesunate (ASAQ)	88.5
Amodiaquine + sulphadoxine-pyrimethamine (AQSP)	85.7
Sulphadoxine-pyrimethamine + artesunate	82.6
Chloroquine+ sulphadoxine-pyrimethamine (CQSP)	72.1
Chloroquine + artesunate	45.3

## Safety and tolerability of artemether/lumefantrine

The safety and tolerability of AL has been confirmed in both adults and children in several studies [10-15]. These data support the use of a six-dose regimen of AL as a safe and well-tolerated treatment in a wide range of patient populations including children.

Safety and tolerability are covered in detail in a companion article in this supplement [55].

## Conclusion

Artemether/lumefantrine has consistently met the WHO criteria for efficacy (>95%) in both adults and children in randomized, controlled trials in malaria-endemic regions [10-15]. Efficacy appeared to be maintained across different populations and regions (Tables 2 and 3); it was unaffected by bodyweight in infants and children and was comparable for dispersible and crushed tablets of AL [14].

Effectiveness studies comparing supervised and unsupervised treatment [44] also demonstrated comparable efficacy data. The 28-day uncorrected cure rate in the per protocol population was also >95% in non-immune adult travellers with *P. falciparum *malaria [15].

A large number of independent studies show the six-dose AL regimen is at least as effective as other artemisinin-based combination therapies and combinations of anti-malarials. A meta-analysis of 32 comparative trials showed AL to be one of the most effective ACTs currently available [54].

The combination of artemether and lumefantrine also has good gametocidal properties, which may help to limit the spread of resistance [10,12]. To date, no resistance to AL *in vivo *has been reported in Africa [1].

## Competing interests

The authors would like to acknowledge that Novartis Pharma AG sponsored this supplement. However, none of the authors works for, or represents in any way, Novartis Pharma AG.

## Authors' contributions

All authors met International Committee of Medical Journal Editors criteria for authorship.
